# Cerebrospinal and Blood Biomarkers in Alzheimer’s Disease: Did Mild Cognitive Impairment Definition Affect Their Clinical Usefulness?

**DOI:** 10.3390/ijms242316908

**Published:** 2023-11-29

**Authors:** Giulia Bivona, Matilda Iemmolo, Giulio Ghersi

**Affiliations:** 1 Department of Biomedicine, Neurosciences and Advanced Diagnostics, University of Palermo, 90127 Palermo, Italy; 2 Department of Biological, Chemical and Pharmaceutical Sciences and Technologies (STEBICEF), University of Palermo, 90128 Palermo, Italy

**Keywords:** biomarkers, flaws, Alzheimer’s disease, amyloid β, phosphorylated tau, CSF, blood

## Abstract

Despite Alzheimer’s Disease (AD) being known from the times of Alois Alzheimer, who lived more than one century ago, many aspects of the disease are still obscure, including the pathogenesis, the clinical spectrum definition, and the therapeutic approach. Well-established biomarkers for AD come from the histopathological hallmarks of the disease, which are Aβ and phosphorylated Tau protein aggregates. Consistently, cerebrospinal fluid (CSF) Amyloid β (Aβ) and phosphorylated Tau level measurements are currently used to detect AD presence. However, two central biases affect these biomarkers. Firstly, incomplete knowledge of the pathogenesis of diseases legitimates the search for novel molecules that, reasonably, could be expressed by neurons and microglia and could be detected in blood simpler and earlier than the classical markers and in a higher amount. Further, studies have been performed to evaluate whether CSF biomarkers can predict AD onset in Mild Cognitive Impairment (MCI) patients. However, the MCI definition has changed over time. Hence, the studies on MCI patients seem to be biased at the beginning due to the imprecise enrollment and heterogeneous composition of the miscellaneous MCI subgroup. Plasma biomarkers and novel candidate molecules, such as microglia biomarkers, have been tentatively investigated and could represent valuable targets for diagnosing and monitoring AD. Also, novel AD markers are urgently needed to identify molecular targets for treatment strategies. This review article summarizes the main CSF and blood AD biomarkers, underpins their advantages and flaws, and mentions novel molecules that can be used as potential biomarkers for AD.

## 1. Background

AD is a neurodegenerative disorder characterized by progressive dysfunction and loss of neurons throughout the brain, which leads to severe cognitive decline and behavioral changes. Being the most frequent form of cognitive decline, AD represents a serious social burden for patients and caregivers and a hard-to-achieve goal for the healthcare system worldwide. Also, an effective treatment for AD is still lacking [1].

While sporadic AD accounts for most of the disease’s cases, familial AD only covers a minority of presentations, with each form having significant aging and genetic risk factors, respectively. Memory and language problems, along with behavioral symptoms, represent the main difficulties AD patients experience across a progressive disease clinical course that is known as the “AD continuum” (see paragraph “How MCI definition affected the studies on CSF AD biomarkers”) [2]. The AD continuum course spans a 15–25-year stretch, starting from a symptom-free period named pre-clinical AD and then continuing as a slight, objective impairment of cognitive functions, which ends in overt dementia [2]. Apart from some exceptions, the Alzheimer’s continuum is the most common form of clinical course in AD presentation [2].

From a histopathological perspective, extracellular deposits of Aβ protein and intracellular accumulation of entangled hyperphosphorylated Tau protein are well-known AD hallmarks. Aβ is a neuronal-derived protein produced through the cleavage of amyloid precursor protein (APP) by ⲁ, β, and ɣ secretase within the endocytic compartment, axons, and dendrites [3,4]. Under normal conditions, Aβ regulates synapse activity by reducing disproportionate synaptic activation to avoid excitotoxicity [5]. However, the overproduction of Aβ decreases the density of dendritic spines, reducing the strength of synaptic transmission, thus leading to synaptic failure [3,5]. Redundant Aβ peptides attach to one another, forming neurotoxic oligomers (dimers, trimers, and tetramers) and fibrils, with the oligomers being the more neurotoxic compound [6]. The Tau protein is a microtubule-associated protein that normally stabilizes the cytoskeleton and maintains the cellular scaffold grip. Modified, misfolded Tau forms, as hyperphosphorylated Tau tends to aggregate and accumulate in the cellular compartment, with dramatic changes in cell organization and morphology due to cytoskeleton detachment [7]. The deposition of intracellular Tau aggregates is not an exclusive characteristic of AD, but rather a common feature among major neurodegenerative disorders and proteinopathies, including progressive supranuclear palsy and dementia with Lewy bodies [8].

From a pathogenic point of view, the amyloid cascade hypothesis posits that Aβ deposition alone primes Tau protein modification, hyperphosphorylation, and accumulation throughout neurons. However, this hypothesis has been progressively set aside, mainly because amyloid-targeting drugs, which counteract Aβ accumulation, have failed to improve AD symptoms and slow down disease progression. According to the amyloid-inflammatory cascade [9,10], the established key event driving the pathobiology of AD consists of three events, with the first being Aβ aggregate extracellular deposition. This gives rise to brain inflammation, also known as neuroinflammation, which causes Tau protein hyperphosphorylation and deposition. The complexity of these sequential phenomena would account for the biochemical and molecular mechanisms responsible for synaptic dysfunction and neuronal loss processes. Beyond these hypotheses, other causative theories underlying AD onset and progression have been postulated, including metabolic dysfunction, vessel pathology, neurotransmission failure, growth factor imbalance, infections, toxins, and inflammation in the brain (so-called neuroinflammation) [11].

The AD diagnostic criteria have profoundly changed over time, thanks to the contribution of the National Institute on Aging–Alzheimer’s Association (NIA-AA), which added biological parameters to the neuropathological, clinical, and neuropsychological ones [12,13]. Biological criteria include fluid and imaging biomarkers to detect the presence of AD as early as possible during the continuum of impairment. Hence, AD diagnosis is made using a combined clinical and biological approach [2]. This approach comes from the efforts of Jack and colleagues, who developed a biomarker-based scheme named ATN to identify AD by detecting Aβ (A = amyloid), phosphorylated Tau (T = phosphorylated Tau), and neurodegeneration (N, defined as measurable total Tau) [13]. Biomarkers of the ATN scheme are divided into neuroimaging and fluid biomarkers. The ATN framework is a research-only framework and signs the line between AD and dementia; indeed, AD clinical presentation within the AD continuum also includes slight cognitive detriment, which is not dementia per se, and, further, non-AD dementia patients never display the presence of both Aβ and phosphorylated Tau [2,14]. Although the ATN scheme is not suitable for clinical practice, it represents a concrete chance to identify AD continuum patients before the onset of overt dementia, and additionally, observational and interventional studies on AD frequently categorize patients based on this approach [15,16].

This review article summarizes the studies on AD biomarkers obtained with a search on Pubmed.com, using the following keywords: “Alzheimer’s Disease”, “biomarkers”, “prediction”, “mild cognitive impairment”, and “measurement”, with a language restriction for English studies and excluding analyses addressing diseases other than cognitive decline. Also, the current article describes the advantages and flaws of AD biomarkers and mentions novel potential biomarkers for AD.

## 2. Current AD Biomarkers: A Short Summary

**Figure 1:**
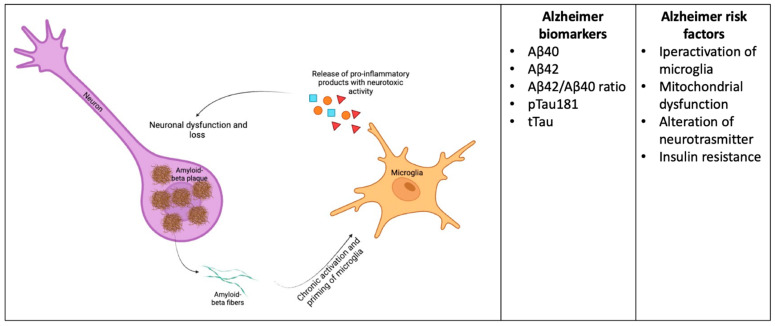
Biomarkers of AD and disease pathomechanisms. Aβ deposition by neurons within the extracellular space can give rise to the chronic activation of microglia, which shifts toward their neurotoxic phenotype, causing neuronal damage. CSF and blood Aβ concentrations, along with pTau and tTau, serve as diagnostic and prognostic biomarkers in subjects having cognitive decline, including mild cognitive impairment and overt AD. Inflammatory molecules produced by activated microglia could be considered as potential biomarkers in these patients, as well as other molecules linked to altered neurotransmission, mitochondria dysfunction, and insulin resistance.

Well-established fluid biomarkers for AD include CSF, plasma, and molecular markers, with CSF Aβ40, Aβ42, Aβ42/Aβ40 ratio, phosphorylated Tau 181 and total Tau (pTau181 and tTau, respectively) being the most used worldwide [17,18,19,20,21,22] (Figure 1). Nonetheless, it should be clarified that these markers present some pitfalls and, on the other hand, that the advancing knowledge around AD pathogenesis legitimates searching for novel molecules. The most attractive field of interest for researchers is represented by molecular and biochemical pathways that have been recently proven to be involved in the core mechanisms of AD pathogenesis [23].

### Microglia Biomarkers

Microglia activation upon stimuli plays a part in AD pathogenesis. Microglia have been deemed to play a fundamental part in determining neuronal injury and loss, thus contributing to the pathophysiology of AD. Microglia are brain-resident immunological cells appointed to prime the inflammatory response against injury. Also, they exert other non-immunological, crucial activities, including modulation of brain function in development and adulthood and regulation of cognition and mood (for more details on non-immunological microglia activities, see refs. [24,25]).

The key role of microglia dysfunction in AD pathogenesis was first uncovered by the observation that some inflammation-related genes are associated with a higher risk of developing AD [26,27]. Also, an increased amount of inflammatory cytokines along with reactive microgliosis has been reported to accompany neuronal injury during AD [28]. Nevertheless, when using the term “dysfunction” in regard to microglia cells, it should be taken into account that it characterizes and summarizes a complex of cellular events rather than a single change or variation in the phenotype and reactivity of these cells. The composite line of heterogeneity among microglia population affects the response of these cells against injuries, along with their capability to regulate specific brain functions [29,30]. Hence, answering the question as to how different subsets of microglia can change upon physiological (genetics, environments, aging, and others) and pathological events (trauma, infections, psychiatric and neurodegenerative diseases), is a pre-requisite to interpreting the role of these cells as cellular mediators in the pathogenesis of brain disease. This is particularly important when considering that some biochemical mechanisms of microglia signaling are being studied as potential targets for neurological diseases, including AD, for which effective treatment is lacking [31]. All the events linking dysfunctional microglia to AD onset and progression are not fully understood. However, three main fixed points on this topic can be stated: (i) microglia can be healthy or unhealthy, based on their subsets and phenotype; unhealthy microglia disrupt brain microenvironment and homeostasis [25,31]; (ii) aberrant microglia phenotypes undergo chronic activation and display neurotoxicity, due to the overproduction of pro-inflammatory molecules and loss of defense capacity [32,33]; (iii) at least one microglia subset, among those that have been described by single-cell RNA sequencing studies, has been proven to drive AD onset [34,35]. It is interesting to note that among the factors influencing microglia dysfunction, a decisive role for aging is recognized, with aging being the main risk factor for sporadic AD.

Given all the above, biomarkers of microglia activation have been evaluated for diagnosing and monitoring AD, with some attracting more attention, including the CX3 chemokine ligand 1 (CX3CL1), triggering receptors expressed in myeloid cells 2 (TREM2), and some metalloproteinases. Studies on CX3CL1 came from observations that the CSF chemokine levels significantly change in AD patients across different stages of the disease, and several investigations on this topic have been performed. Unfortunately, findings have been controversial and difficult to interpret, also due to an ambiguous activity of CX3CL1 toward amyloid deposition and Tau phosphorylation [24,36]. While the role of microglia as biomarkers in AD has been partially discovered, and some difficulties in entering clinical practice are apparent for these molecules (clinical usefulness over current biomarkers is far from being demonstrated, requiring high-cost investigation and validation studies), conversely, it seems more appropriate for these molecules to be used as treatment targets in AD [31] (Table 1).

## 3. Advantages and Flaws of AD Current Biomarkers

The main advantages of AD CSF classical biomarkers include the standardization process of instrumentations, procedures, and materials for their measurement, which aligns the results among diverse laboratories worldwide. Also, a quality program has been set up, assuring homogeneity and high-performance analyses [37,38,39]. Finally, pre-analytical issues have been fully overcome in regard to, for instance, the tendency of CSF proteins like Aβ to attach to plastic, which led to an underestimated marker concentration due to the adhesion of proteins to tubes for collection or storage. Using tubes that prevent adsorption (e.g., polypropylene tubes) or serially determining the CSF Aβ42/Aβ40 ratio helps minimize the impact of using improper tubes on the interpretation of the results [2]. The disadvantages of CSF classical biomarkers can be summarized as follows: firstly, the procedure is invasive, hampering serial measurement and the evaluation of the kinetics. The lack of repeatability also hinders following the progression of the disease and weakens the findings on the eventual prediction power of biomarkers. Another difficulty refers to the low specificity of certain markers. Tau protein, for instance, is altered in various neurodegenerative diseases and proteinopathies, and studies have demonstrated that AD patients with established diagnoses present low expression of Tau proteins compared to non-AD patients [24]. Further, it should be taken into account that phosphorylation in different sites of many proteins makes them act in different ways. Various forms of the Tau protein can be measured in AD patients (pTau 181, pTau 231), but no clear knowledge has been reached on the differential significance of these forms. Finally, Aβ could be the most specific biomarker for AD, but amyloidosis patients share the same Aβ alterations as those with AD (Table 1).

The advantages of CSF Aβ40, Aβ42, the Aβ42/Aβ40 ratio, pTau181, and tTau have been largely documented through a broad body of data. Unfortunately, the studies performed to obtain these data displayed some pitfalls, which compromise the validity of the results obtained. Common pitfalls of the studies performed include diversity among statistics, marked discrepancies among the cut-offs used for measurement, and small sample size. However, the core flaw of the investigations on this topic could consist of the clinical criteria used for the enrollment of patients, generating a bias intrinsic to the study groups, which lies in the MCI definition itself [40]. Namely, the MCI definition has been used to discriminate between subjects with moderate cognitive impairment and those having AD, whereas clear discrimination in the real world, at least from a clinical perspective, does not exist. The fact that the MCI definition is slightly forced and inconsistent in practice is proven by the circumstance that the most recent studies on this topic bypassed such a definition utilizing the patient’s categorization criteria [41].

### Flaws of the Studies on MCI and AD Biomarkers

Between 1999 and 2004, the MCI definition as a nosological entity was formulated for the first time and then revised [41,42], specifying the diagnostic tools to be used for the evaluation of the domains that are either affected or preserved during MCI. Shortly, these criteria include subjective memory impairment, objective memory deficiency, cognition impairment, auto-sufficiency in daily activities, and preserving mental health. However, the MCI definition progressively underwent various changes over time. According to the current definition, MCI is regarded as a syndrome characterized by cognitive decline with three possible destinies: it can remain stable over time, or it can evolve into AD, and an evolution toward non-AD dementia forms is possible. In the case that the MCI transforms into AD, it belongs as a phase of the continuum of AD, and it is named pre-AD MCI [43]. Note that no fixed transition points along the route from MCI to AD can be identified, and it is widely accepted that no transition steps connecting the asymptomatic, pre-dementia, and overt dementia phases can be analyzed or established [12,44]. Also, in the very early stage of the disease, it is challenging to distinguish the AD continuum presentation from age-related cognitive impairment [43], making it hard for clinicians to categorize subjects according to the type and entity of their cognitive impairment. As a consequence, it is very challenging to include the continuum of impairment within the research and clinical setting, as it was prophetically presaged by the NIA-AA workgroups on diagnostic guidelines for Alzheimer’s disease [43] because it is hard to identify and distinguish among asymptomatic and symptomatic pre-AD MCI, MCI, and aging-related decline. Almost all the studies on biomarkers for AD were performed on patients having different cognitive impairment types, which were generically categorized as MCI. Such patient stratification seems to be at least debatable. Studies addressing the question as to whether traditional biomarkers are able to predict the onset of AD in individuals with MCI [45,46,47,48,49] had the most serious consequences of this bias, influencing the results of the studies performed and distorting our knowledge around the effective prediction capability of CSF biomarkers. In this context, all the studies reporting the Aβ42/Aβ40 ratio to be a reliable predictor of MCI progression toward AD suffer from an uncertain categorization of subjects. Indeed, most of the analyses carried out to compare the CSF Aβ42/Aβ40 ratio between MCI and AD patients defined the MCI group as a hodgepodge of para-physiological (age-related decline) and pathological conditions (prodromal non-AD dementia), instead of including exclusively pre-AD MCI [46,47,48,49].

All the studies performed before Albert’s criteria definition suffer from this bias, and several studies that have been performed after the MCI definition by Albert used the first Petersen’s criteria [48] as a methodology for selecting patients as well. Nevertheless, it is worth mentioning the elegant study [50] by Baldeiras et al., which presents a very accurate selection of the study populations due to strict and updated inclusion criteria for the MCI group (Table 2).

Collectively, it could be said that MCI has long had a vague definition, counting any kind of physiologic and pathologic conditions, including age-related impairment, non-AD-related impairment, and pre-AD impairment. Also, subjective self-reported complainers have been enrolled in most of the analyses performed, with no long follow-up period documenting their evolvement in AD. Hence, findings from the studies carried out on such a melting pot of patients should be taken with a grain of salt, and several analyses on the predictive value of AD biomarkers in pre-AD MCI should be performed again.

Another major flaw affects most of the studies carried out in demented and non-demented patients to measure CSF biomarkers. The literature of the last decade often proposes a sort of refrain occurring in almost any study. The early identification of MCI would be particularly important to slow down or stop the progression of the disease, based on the growing knowledge and fast development of new, disease-modifying treatment approaches. Although this is theoretically true, no effective treatment for AD is available so far, despite several attempts, and no impact in clinical practice can be detected among these studies [44]. From a therapeutic perspective, the search for good biomarkers can help evaluate their use as an endpoint in clinical trials. Nonetheless, if a study is conceived out of concrete basis, supposing one day an unknown treatment will be available, that study is not sufficient to define or establish fixed points within a certain topic, remaining speculative.

## 4. Novel AD Biomarkers

CSF biomarker measurement displays the following other pitfalls: high costs to perform the analyses, time-consuming analyses, and difficulty in serial sampling repetition [54,55]. The obvious advantages of plasma sample collection over CSF ones help us to understand the profuse efforts to identify blood-based biomarkers. The search for the appropriate methodologies and molecules has been long, and controversial results have been attained, with their reliability in clinical practice needing to be further confirmed. Among all, most studied plasma biomarkers include CSF classical biomarkers (Aβ42, Aβ40, Aβ42/Aβ40 ratio, pTau181, pTau217, pTau231), neurofilament light chain, neurogranin, neuronal-derived exosomes, neuronal-enriched extracellular vesicles, glial acid fibrillary protein (GFAP), triggering receptors expressed in myeloid cells 2 (TREM2), monocyte chemoattractant protein 1 (MCP1), and some pro-inflammatory and anti-inflammatory cytokines, including interleukin (IL)-1β, IL-2, IL-6, IL-10, and IL-18 [41,54,55].

AD classical biomarkers (Aβ42, Aβ40, Aβ42/Aβ40 ratio, and pTau181) have been measured in AD patients’ plasma and in that of subjects with mild cognitive decline [51,52,53,56,57,58,59]. In some cases, the analyses were performed on small study groups with short follow-up periods, limiting the strengths of the results obtained, although patients’ categorization as cognitively unimpaired with and without increased Aβ levels in the CSF overcame MCI definition-related bias [41].

Studies on large cohorts of patients (>1000) [46,50] have been carried out as well, with some authors reporting classical biomarkers to have impressive diagnostic power [52,56,60]. However, some considerations should be taken into account. First, several discrepancies in the trend of plasma levels of Aβ42 and Aβ40 have been shown, with some analyses finding plasma Aβ42 and Aβ40 to increase in the early stage of AD, while other investigations revealed a decrease [61,62]. Second, no difference in Aβ42 and Aβ40 plasma levels between AD and non-AD patients were reported by Pannee et al., Olson et al. and others [39,63,64], as well as no difference between MCI patients developing AD and those who did not documented in the studies by Mahaman et al. and Blennow et al. [54,65]. Even when an excellent performance of classical biomarkers was reported, some concerns still remained. For instance, Karakiri et al. performed analyses on four cohorts of patients (*n* = 1131), reporting plasma pTau 181 to progressively increase during the AD continuum. However, the analyses on the primary cohort only revealed that plasma pTau displayed an excellent performance when comparing AD patients to young adults but not to MCI patients [51]. Discrepancies among the results can be explained by several factors, including molecule-linked factors (Aβ can be released by organs other than the brain), technical issues (plasma proteins can bind Aβ peptides interfering with the analytical procedure), and, to a small extent, by individual factors (racial/ethnic differences) [60,64]. Third, huge criticism is related to the standardization process for the plasma level measurement of the biomarkers. Aligning results among laboratories by using instrumentations, procedures, and materials certified by a harmonization process and a Quality Control Program is necessary [60,66,67].

New technologies allowing the measurement of very low concentrations of biomarkers in blood have been developed, with pTau plasma level single molecule array technology [68].

Plasma neurofilament light chains (NfLs), neuronal-derived exosomes, and neuronal-enriched extracellular vesicles are interesting molecules to be used as potential biomarkers for AD as well [69]. NfLs were initially considered good biomarkers for AD, as some studies provided evidence that they can predict AD onset and distinguish AD from non-AD patients [70]. However, nowadays, they are regarded as markers of multiple neurodegenerative diseases rather than specific AD biomarkers [71]. A very recent study performed using machine learning models investigated the NfL capability of AD prediction in combination with other variables, including sociodemographic and self-reported health information. Jia et al. showed NfLs to be good predictors of AD together with age, blood Tau protein, and education level [69], but reported some other molecules (taurine, inosine, xanthine, and glutamine) to display a good prediction potential for AD. Johansson et al. analyzed a small cohort of familial AD patients, reporting plasma NfL levels to change over time while progressing from the pre-symptomatic phase to the clinical one across a 10-year period [71]. Nevertheless, the authors reported that GFAP and pTau plasma levels changed significantly earlier than NfLs in the same time span, with GFAP being the first and pTau the second in the order of biomarker changes over time [71].

Several neuronal damage-linked molecules have been recognized as having diagnostic accuracy for AD, including neurogranin, synaptotagmins, and neuronal calcium-sensing protein VLP1, with neurogranin showing the most encouraging potentiality [2,72].

Other molecules that are currently under investigation could be considered valuable biomarkers for AD, with inflammation- and microglia-related markers being good candidates either to identify the presence of AD or search for novel molecular targets for treatment approaches [54,58]. However, more investigations are required to prove the eventual role of these molecules as biomarkers and therapeutic targets.

Finally, proteomic and metabolic approaches for identifying AD biomarkers are currently being investigated [55,73].

## 5. Conclusions

A biomarker shows clinical usefulness if it is able to modify a patient’s clinical management by diagnosing the disease, predict progression toward more severe conditions, and control the response to treatment. In this scenario, CSF biomarkers for AD are good indicators of the presence of AD in cognitively declined subjects, even more so when they are used to distinguish AD-related dementia from other, non-AD-related forms of dementia. In non-demented patients with moderate impairment of cognition, CSF biomarkers for AD might help identify patients who are more likely to develop AD, but their prediction potential is limited. This is also because of the bias affecting the studies carried out on this topic, whose results should be taken with a grain of salt. Above all, a main flaw has been poorly described regarding the MCI definition, which has been, for a long time, a sort of melting pot of both physiologic and pathologic conditions (including age-related cognitive impairment, non-AD cognitive decline, or prodromal AD). Since a more precise characterization of AD-related impairment has been proposed, according to more precise and novel criteria, a few studies described the CSF and plasma levels of pre-AD and AD biomarkers, but studies performed on large samples and long follow-up periods are lacking. Enlarging cohorts of patients and controls, ensuring strictly established inclusion criteria for the enrollment, and extending the follow-up would give strength to the results obtained in this first end of investigations, representing a future research hint in this field. Indeed, the selection of appropriate biomarkers in established pathological conditions is a key point for using them as an endpoint in clinical trials, helping the search for effective treatments. The studies in this research area should describe a very precise category of patients, avoiding subjective inclusion criteria for enrollment as much as possible, and following them up for a long period (>15 years), to establish the difference (if a difference exists) between mild cognitive decline and AD in terms of Aβ and Tau levels.

Further, the development of valuable methods and definitive standardization of blood-based biomarkers is urgently needed to reduce the high costs related to the measurement of CSF markers and the use of imaging tools, enhancing the repeatability of measurements and patient comfort.

## Figures and Tables

**Table 1:** Advantages and flaws of classical and novel AD biomarkers.

Biomarkers	Advantages	Flaws
Classical CSF biomarkers (CSF Aβ40, Aβ42, Aβ42/Aβ40 ratio, pTau181 and tTau)	Standardization process; quality program for harmonization; strong correlation with pathomechanisms	Invasive; poor repeatability; relatively small specificity (pTau and Tau)
Microglia biomarkers	Strong correlation with pathomechanisms	No validation studies; poor specificity
Blood classical biomarkers (plasma Aβ40, Aβ42, Aβ42/Aβ40 ratio, pTau181 and tTau)	Easy to perform; repeatability; not invasive	No validation studies; no clear clinical usefulness
Blood novel biomarker (NfL, neuronal-derived exosomes, and neuronal-enriched extracellular vesicles)	Repeatability; not invasive	Poor evidence on their clinical usefulness; no specificity; further studies required

**Table 2:** Main findings from the studies performed on AD biomarkers in cognitive declined patients.

Author	Cohort Characteristics	Sample Size	Main Findings	Year	Ref.
Hansson et al.	Stable MCI, pre-AD MCI	137	Abeta42/Abeta40 ratio is a predictive biomarker for AD	2007	[46]
Hertze et al.	Stable MCI and AD	260	Aβ42 and Tau have low predictive value for AD diagnosis	2010	[47]
Frolich et al.	Stable MCI, pre-AD MCI, and AD	115	Combination of Aβ42/Aβ40, pTau, and tTau has best value for AD than tTau alone and hippocampal volume	2017	[49]
Baldeiras et al.	AD and non-AD based on ATN scheme	1128	Aβ-related markers are the best predictors of AD	2022	[50]
Chatterjee et al.	Cognitively unimpaired subjects	100	Plasma GFAP, pTau181, pTau231, and NFL correlate with cognition, but not with hippocampal volume.	2022	[41]
Karikari	AD continuum	1131	pTau predicts AD along the AD continuum	2020	[51]
Janelidze	AD, MCI and cognitively unimpaired patients	589	Plasma Tau predicts AD in cognitively unimpaired patients	2020	[52]
Chen	MCI	22	Aβ42 and Tau are good predictors of AD development in MCI subjects	2019	[53]
Smirnov	MCI, dementia	312	plasma PTau181 and 231 are good predictors of AD	2022	[14]
Bateman	Autosomal AD mutation carriers	128	CSF Aβ42 and Tau decline 25 and 15 years before symptom onset, respectively.	2012	[21]
Stevenson-Hoare	AD	1439	Aβ40/Aβ42 ratio, GFAP, and NfL display the best prediction accuracy for AD diagnosis	2023	[15]

## Abbreviations

Alzheimer’s Disease (AD); cerebrospinal fluid (CSF); Mild Cognitive Impairment (MCI); Amyloid β (Aβ); amyloid precursor protein (APP); National Institute on Aging–Alzheimer’s Association (NIA-AA); phosphorylated Tau (pTau); total Tau (tTau); CX3 chemokine ligand 1 (CX3CL1); triggering receptors expressed in myeloid cells 2 (TREM2); plasma neurofilament light chain (NfL).
